# Incidence of hepatitis C virus infection in the prison setting: The SToP‐C study

**DOI:** 10.1111/jvh.13895

**Published:** 2023-11-07

**Authors:** Behzad Hajarizadeh, Joanne M. Carson, Marianne Byrne, Jason Grebely, Evan Cunningham, Janaki Amin, Peter Vickerman, Natasha K. Martin, Carla Treloar, Marianne Martinello, Andrew R. Lloyd, Gregory J. Dore, the SToP‐C study group, Stuart Loveday, Nicky Bath, Tony Butler, Georgina Chambers, Roy Donnelly, Colette McGrath, Julia Bowman, Lee Trevethan, Katerina Lagios, Luke Grant, Terry Murrell, Victor Tawil, Annabelle Stevens, Libby Topp, Alison Churchill, Kate Pinnock, Steven Drew, Mary Harrod, Pip Marks, Mahshid Tamaddoni, Stephanie Obeid, Gerard Estivill Mercade, Maria Martinez, William Rawlinson, Malinna Yeang, Matthew Wynn, Christiana Willenborg, Angela Smith, Ronella Williams, Brigid Cooper, Kelly Somes, Carina Burns, Camilla Lobo, Karen Conroy, Luke McCredie, Carolyn Café, Jodie Anlezark

**Affiliations:** ^1^ The Kirby Institute University of New Soth Wales (UNSW) Sydney New South Wales Australia; ^2^ Faculty of Medicine and Health Sciences Macquarie University Sydney New South Wales Australia; ^3^ Population Health Sciences University of Bristol Bristol UK; ^4^ Division of Infectious Diseases & Global Public Health University of California San Diego San Diego California USA; ^5^ Centre for Social Research in Health University of New Soth Wales (UNSW) Sydney New South Wales Australia

**Keywords:** HCV, intravenous substance abuse, opiate substitution treatment, cohort study, correctional facilities

## Abstract

People in prison are at high risk of HCV given high injecting drug use prevalence. This study evaluated HCV incidence and associated injecting drug use characteristics in prison. The SToP‐C study enrolled people incarcerated in four Australian prisons. Participants were tested for HCV at enrolment and then every 3–6 months (October‐2014 to November‐2019). Participants eligible for this analysis included those at‐risk of HCV primary infection (anti‐HCV negative) or re‐infection (anti‐HCV positive, HCV RNA negative) with follow‐up assessment. A total of 1643 eligible participants were included in analyses (82% male; median age 33 years; 30% injected drugs in prison; 1818 person‐years of follow‐up). Overall HCV incidence was 6.11/100 person‐years (95%CI: 5.07–7.35), with higher rate of re‐infection (9.34/100 person‐years; 95%CI: 7.15–12.19) than primary infection (4.60/100 person‐years; 95%CI: 3.56–5.96). In total population (*n* = 1643), HCV risk was significantly higher among participants injecting drugs in prison [vs. no injecting; adjusted hazard ratio (aHR): 10.55, 95%CI: 5.88–18.92), and those who were released and re‐incarcerated during follow‐up (vs. remained incarcerated; aHR: 1.60, 95%CI: 1.03–2.49). Among participants who injected recently (during past month, *n* = 321), HCV risk was reduced among those receiving high‐dosage opioid agonist therapy (OAT), i.e. methadone ≥60 mg/day or buprenorphine ≥16 mg/day, (vs. no OAT, aHR: 0.11, 95%CI: 0.02–0.80) and increased among those sharing needles/syringes without consistent use of disinfectant to clean injecting equipment (vs. no sharing, HR: 4.60, 95%CI: 1.35–15.66). This study demonstrated high HCV transmission risk in prison, particularly among people injecting drugs. High‐dosage OAT was protective, but improved OAT coverage and needle/syringe programmes to reduce sharing injecting equipment are required.

## 1 INTRODUCTION

Injecting drug use is the primary mode of hepatitis C virus (HCV) transmission across the world, particularly in high‐income countries.^1^, ^2^ People who inject drugs are over‐represented in prisons in most countries, primarily for drug‐related offences,^3^ with an estimated 58% of people who inject drugs having an incarceration history globally.^4^ People in prison are at increased risk of HCV infection given high prevalence of injecting drug use and limited access to harm reduction services [e.g. opioid agonist therapy (OAT) and needle and syringe programmes].^5^ In 2014, an estimated 10 million people in prisons worldwide were living with HCV infection.^6^ Despite the high HCV burden in the prison setting, data regarding HCV incidence are limited,^7^ primarily due to logistical difficulties of implementing prospective studies with longitudinal follow‐up in prisons. Most available prison‐based cohort studies evaluating HCV incidence are old or limited by small study population size, or short follow‐up.^7^, ^8^, ^9^, ^10^, ^11^

The Surveillance and Treatment of Prisoners with hepatitis C (SToP‐C) study evaluated the feasibility and effectiveness of direct‐acting antiviral (DAA) treatment scale‐up in prisons, and demonstrated a significant reduction in HCV incidence in the period following DAA treatment scale‐up.^12^ The present analysis evaluated the overall HCV incidence in SToP‐C prisons independent of the study period, including detailed analyses of characteristics of injecting drug use associated with the risk of HCV infection.

## 2 METHODS

### 2.1 Study setting and design

The SToP‐C study was a non‐randomised clinical trial within a longitudinal cohort of participants enrolled to evaluate the effectiveness of HCV treatment‐as‐prevention in the prison setting. The detailed methodology has been described previously.^12^ In brief, the study was conducted between October 2014 and November 2019 in four prisons in New South Wales (NSW), Australia. Study prisons included two male‐only maximum‐security prisons (Goulburn and Lithgow), and two medium‐security prisons, including one male (Outer Metropolitan Multi‐Purpose Correctional Centre; OMMPCC), and one female (Dillwynia) prison. Harm reduction services available in the NSW prisons include OAT, with eligibility based on a clinical evaluation for opioid dependence and the capacity of the programme, determined by the prison health services.^13^ In addition, Fincol (a disinfectant containing quaternary ammonium compound) is available to all inmates to clean injecting apparatus.^14^ No needle and syringe programmes are available in Australian prisons.

In the initial phase of the study (October 2014 to mid‐2017), prison health services offered HCV treatment as standard of care, with interferon‐based treatment (October 2014 to March 2016) followed by DAA treatment (March 2016 onward). In the second phase of the study (mid‐2017 to November 2019), rapid scale‐up of DAA treatment was initiated. In both phases, HCV awareness campaigns were held for people in prison, prison officers and prison health care staff. Dedicated hepatitis nurses and prison officers were funded through the study to increase the capacity of prison hepatitis services to facilitate DAA treatment scale‐up.

### 2.2 Study participants and assessments

All people incarcerated in SToP‐C prisons who were 18 years or older were eligible for enrolment. People were excluded if they did not have adequate English proficiency to provide informed consent, or if they were housed with a very high security designation, making clinic attendance for study visits logistically difficult.

At enrolment, participants were tested for anti‐HCV (ARCHITECT Anti‐HCV, Abbott, USA and Murex Anti‐HCV, DiaSorin, Italy), and those with a positive result were tested for HCV RNA (COBAS TaqMan, Roche, Switzerland; lower limit of detection 15 IU/mL). Participants with an HCV RNA positive result (i.e. current HCV infection) were offered HCV treatment through the prison health service in the first phase and through the SToP‐C study in the second phase. Participants included in this analysis were those with an anti‐HCV negative result (i.e. uninfected and never exposed) who were therefore at‐risk of HCV primary infection and those with an anti‐HCV positive and an HCV RNA negative result (i.e. previously infected) who were therefore at‐risk of HCV re‐infection. These participants, if stayed in SToP‐C prisons, were followed and assessed by anti‐HCV and/or HCV RNA testing at scheduled visits every 3–6 months. Follow‐up assessment for those who transferred to other prisons or released to freedom was conducted at any time when they returned to SToP‐C prisons.

Participants who were HCV RNA positive (i.e. HCV infection) at enrolment or during follow‐up, and subsequently cleared HCV (either through treatment or spontaneously) were at risk of HCV re‐infection following HCV clearance and re‐entered the follow‐up. Participants who were transferred to a non‐SToP‐C prison, or released to freedom during follow‐up, could re‐enter the study if they returned into a SToP‐C prison. The study enrolment continued from October 2014 to September 2019, with final follow‐up in November 2019.

At enrolment, demographic, clinical and risk behavioural data were collected through a structured questionnaire‐based interview by the study nurses. Risk behaviour questions were about drug use behaviours (e.g. injecting and non‐injecting drug use, drug type, frequency of drug use, sharing needle and syringes, using disinfectant to clean injecting equipment and OAT use) and non‐drug use related HCV risks (e.g. tattooing, piercing or fights in prison). The risk behaviour interview was repeated at all follow‐up visits.

### 2.3 Study outcome

HCV incidence was the primary study outcome, and incidence of HCV primary infection and re‐infection was the secondary outcomes. Incident HCV primary infection was defined as a positive anti‐HCV among participants with a negative anti‐HCV at the previous visit. Incident HCV re‐infection was defined as a positive HCV RNA among participants with a negative HCV RNA and positive anti‐HCV at the previous visit. Among participants receiving HCV treatment, re‐infection following treatment was defined as a recurrent positive HCV RNA after the end of treatment in combination of either a heterologous HCV strain from the primary infecting strain (if pre‐treatment sample available), or any recurrent positive HCV RNA test after achieving sustained virological response (SVR12; if pre‐treatment sample not available). The date of incident HCV infection was estimated as mid‐point between last HCV negative and first HCV positive test. Anti‐HCV test and HCV RNA test were considered for estimated date of primary infection, and re‐infection, respectively.

### 2.4 Statistical analysis

The analysis population included participants who were at risk of HCV infection (primary or re‐infection) at enrolment or during the study with at least one follow‐up assessment. The incidence of HCV infection (primary infection, re‐infection and combined) and corresponding 95% confidence intervals (CI) was calculated as rates per 100 person‐years. Poisson distribution was used for incidence analysis, with follow‐up censored at estimated date of incident HCV infection, or last follow‐up assessment. Last follow‐up assessment included the study visit before prison transfer or release to freedom (where no return was documented), or the last study visit before study closure.

Two stratified analyses were conducted. In the first analysis, HCV incidence was evaluated in two groups based on incarceration status during follow, including: (i) participants who were incarcerated for the entire follow‐up period; and (ii) those who were released and re‐incarcerated during follow‐up.

In the second stratified analysis, HCV incidence was evaluated in three risk groups based on injecting drug use at the beginning of follow‐up, including participants who: (i) had never injected; (ii) had a history of injecting but not during their current imprisonment; and (iii) injected during their current imprisonment. Cumulative HCV incidence was assessed using the Kaplan–Meier method and compared across the risk groups using log‐rank test.

Cox proportional hazard regression analyses were used to evaluate factors associated with HCV infection in two analysis populations. In the first analysis, all participants at‐risk of HCV infection were included. Hypothesised covariates were determined a priori and included age, sex, Australian Indigenous ethnicity (Aboriginal and/or Torres Strait Islander), prison site, duration of imprisonment, incarceration status during follow‐up, HCV testing interval during follow‐up, tattoo or piercing in prison, injecting drug use in the current imprisonment and OAT use. Collinearity existed between sex and prison site (three male‐only and one female‐only prisons) and between injecting drug use and OAT use (data of OAT were only collected among those with a history of past or current injecting). Three separate adjusted models were developed, each including variables with no collinearity.

In the second analysis, only participants who reported injecting drugs in the past month in prison were included. This analysis was conducted to evaluate characteristics of injecting drug use associated with HCV infection. Injecting drug use related covariates in this analysis were determined a priori and included frequency of injecting, drug type injected, use of a new sterile needle and syringe, use of disinfectant for cleaning needles and/or syringes, and OAT use. OAT dosage were categorised as ‘high’ (methadone ≥60 mg/day; buprenorphine ≥16 mg/day) and ‘low’ (methadone <60 mg/day; buprenorphine <16 mg/day), based on Australian and international clinical guidelines.^15^, ^16^ All these covariates referred to participant's behaviour during the past month to minimise recall bias.

Across all analyses, the intervention in the original SToP‐C study (HCV treatment scale‐up in the second phase) was accounted for all models by inclusion of treatment scale‐up period as a covariate. All risk behaviour variables were included as time‐varying in the models. Covariates with a *p* value <.20 in unadjusted models were included in the adjusted models, with the final models including all variables eligible for inclusion in adjusted analyses (no stepwise methods were used). Statistical significance was assessed at *p* < .05 (two‐sided *p* values). Data analysis was performed using Stata 17.0 (StataCorp, College Station, TX, USA).

All participants provided written informed consent before study procedures. The study protocol was approved by NSW Justice Health and Forensic Mental Health Network Human Research Ethics Committee (HREC/14/JH/7), Aboriginal Health and Medical Research Council Human Research Ethics Committee (1047/14 and 1253/17) and NSW Corrective Services Ethics Committee. The study was conducted according to the Declaration of Helsinki and International Conference on Harmonization Good Clinical Practice (ICH/GCP) guidelines, and registered with clinicaltrials.gov (NCT02064049).

## 3 RESULTS

### 3.1 Participant characteristics

**FIGURE 1:**
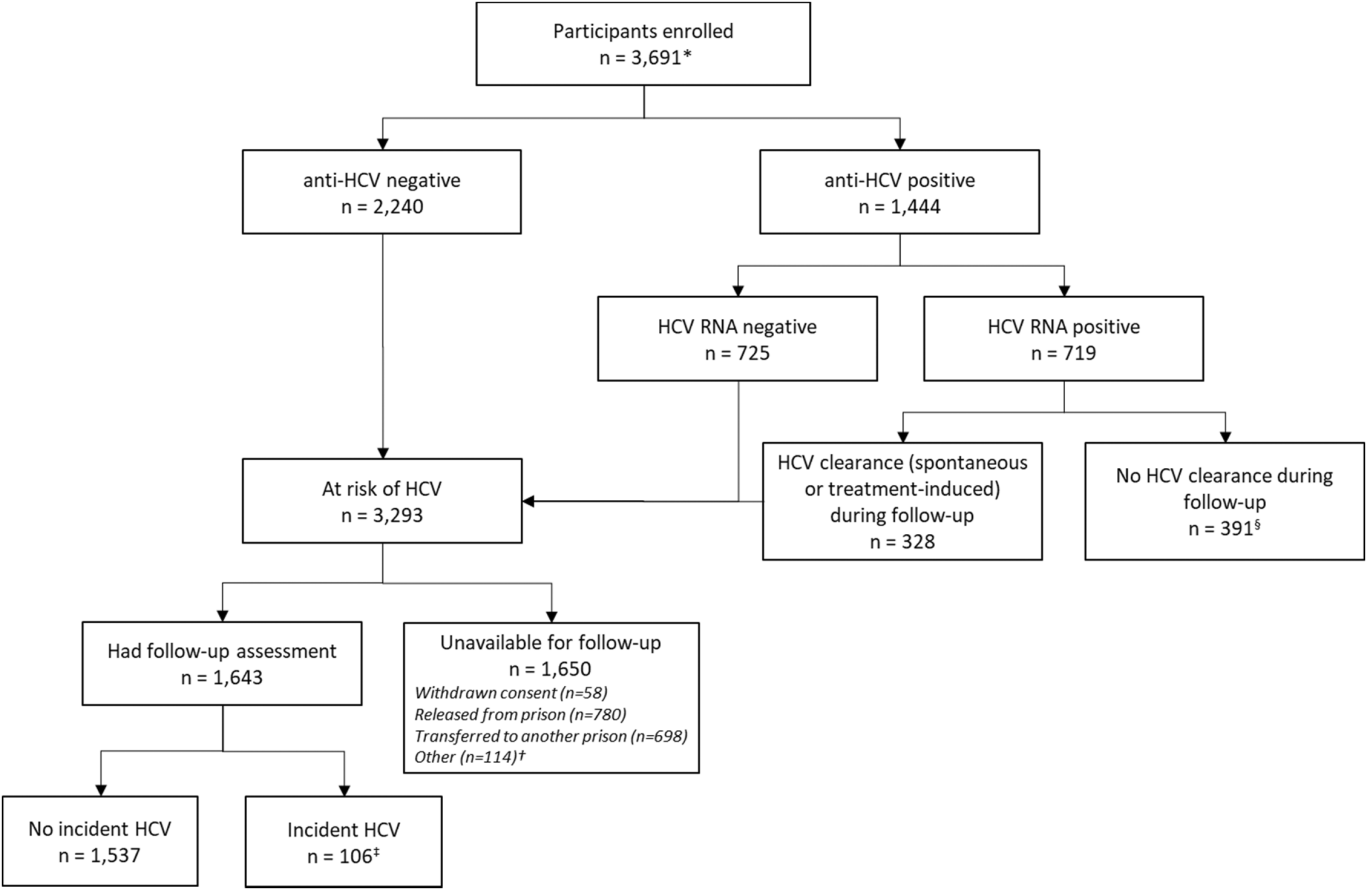
Overview of the study population. * HCV test results at enrolment were not available for seven participants. † Most (*n* = 104) are participants who were enrolled during late 2019 and were not due for follow‐up or there was no access to the participant by the end of the study. ‡ Five participants had the second incident HCV infection after clearance (total HCV incident events = 111). § Included participants who did not receive treatment, or those who received treatment but did not have any record of post‐treatment follow‐up or clearance.

A total of 3691 participants were enrolled, representing 89%, 80%, 62% and 53% of all people incarcerated in OMMPCC, Dillwynia, Lithgow and Goulburn prisons by September 2019. An overview of the study population at enrolment and during follow‐up is illustrated in Figure 1. At enrolment, 2240 participants were at‐risk of primary HCV infection (anti‐HCV negative), 725 participants were at‐risk of HCV re‐infection (anti‐HCV positive and HCV RNA negative) and 719 participants had HCV infection (HCV RNA positive). No participant had HIV, while six had chronic hepatitis B. Among 719 participants with HCV infection at enrolment, 328 participants cleared the infection during the follow‐up. Thus, a total of 3293 participants were at‐risk of HCV infection at enrolment or during the study (89% of total population enrolled), among whom 1643 participants (50%) had at least one follow‐up visit and were included in this analysis (Figure 1). Among participants unavailable for follow‐up, the most common reasons were release from prison or transfer to another prison (90%; *n* = 1478/1650).

Among the analysis population (*n* = 1643), 82% were male, the median age was 33 years, and the median duration of imprisonment was 17 months. At the beginning of follow‐up, 47% of participants (*n* = 776/1643) reported a history of injecting drug use, including 30% (*n* = 487/1643) reporting injecting drugs during the current imprisonment, and 20% (*n* = 321/1643) reporting injecting drugs in the past month in prison. Among participants who reported injecting drugs during the current imprisonment (*n* = 487), 26% were currently receiving OAT (18% receiving high‐dose OAT). Among those reporting injecting drugs in the past month (*n* = 321), 49% reported injecting once a day or more frequently, 82% reported injecting methadone, buprenorphine or other opiates, and 84% reported using a shared needle or syringe while in prison (Table 1). Most demographic and behavioural characteristics were comparable between participants with follow‐up (the analysis population) and those without follow‐up. However, those with follow‐up had a longer duration of imprisonment, with a lower proportion being on remand (i.e. unsentenced), and a higher proportion receiving OAT (Table 1).

**TABLE 1:** Background and behavioural characteristics of the SToP‐C study participants at risk of HCV infection at enrolment, by follow‐up assessment.

	Had no follow‐up assessments (*n* = 1650)	Had at least one follow‐up assessment (*n* = 1643)
Prison site, *n* (%)		
Goulburn	494 (30)	497 (30)
Lithgow	430 (26)	452 (28)
OMMPCC	395 (24)	401 (24)
Dillwynia	331 (20)	293 (18)
Gender, *n* (%)		
Female	331 (20)	291 (18)
Male	1319 (80)	1350 (82)
Transgender	0 (0)	2 (<1)
Age (year), median (Q1, Q3)	32 (26, 39)	33 (27, 42)
Aboriginal and or Torres Strait Islander, *n* (%)		
No	1130 (68)	1192 (73)
Yes	445 (27)	426 (26)
Not available	75 (5)	25 (2)
Country of birth, *n* (%)		
Australia	1287 (78)	1273 (78)
Other countries	290 (18)	343 (21)
Data not available	73 (4)	23 (1)
Formal education level, *n* (%)		
No formal education or completed primary school	505 (31)	548 (33)
Completed high school	769 (47)	780 (47)
Tertiary education	299 (18)	284 (17)
Data not available	77 (5)	31 (2)
Duration of stay in the current prison (month), median (Q1, Q3)	5 (2, 17)	17 (5, 42)
Sentenced, *n* (%)		
No (on remand)	564 (34)	361 (22)
Yes	1014 (61)	1259 (77)
Not available	72 (4)	23 (1)
Previous imprisonment, *n* (%)		
No	428 (26)	506 (31)
Yes	1150 (70)	1114 (68)
Data not available	72 (4)	23 (1)
Tattoo or piercing in prison (current imprisonment), *n* (%)		
No	1510 (92)	1552 (95)
Yes	7 (4)	62 (4)
Data not available	70 (4)	29 (2)
Injecting drug use status, *n* (%)		
Never injected	787 (48)	847 (51)
Had history of injecting, but not in the current imprisonment	373 (23)	289 (18)
Injected longer than 6 months ago (current imprisonment)	45 (3)	75 (5)
Injected in the previous 2–6 months (current imprisonment)	65 (4)	91 (6)
Injected in the previous month (current imprisonment)	311 (19)	321 (20)
Data not available	69 (4)	20 (1)
Opioid agonist therapy^a^, *n* (%)		
Never	252 (60)	239 (48)
Yes, previously	108 (26)	128 (26)
Yes, currently	63 (15)	126 (26)
Currently receiving opioid agonist therapy^a^, *n* (%)		
No	360 (85)	367 (74)
Yes, low dosage	22 (5)	33 (7)
Yes, high dosage	41 (10)	91 (18)
Yes, dosage data not available	0	2 (<1)
Frequency of injecting^b^, *n* (%)		
Less frequently than once a week	81 (26)	75 (23)
1 to 6 days per week, not daily	74 (24)	79 (25)
Once a day or more	156 (50)	158 (49)
Data not available	0 (0)	9 (3)
Substance injected^b^, *n* (%)		
Heroin	51 (16)	52 (16)
Methadone or buprenorphine	279 (90)	263 (82)
Other opiates (e.g. codeine, pethidine, opium, morphine and oxycodone)	5 (2)	8 (2)
Methamphetamine	111 (36)	144 (45)
Cocaine	0 (0)	4 (1)
Other substances	2 (1)	2 (1)
Substance injected most frequently^b^, *n* (%)		
Heroin	11 (4)	8 (3)
Methadone, buprenorphine, or other opioids	265 (85)	250 (78)
Methamphetamine	28 (9)	53 (17)
Other substances	1 (<1)	0 (0)
Data not available	6 (2)	10 (3)
Used a new sterile needle and syringe for injecting drugs^b^, *n* (%)		
Yes, for all injections	14 (5)	10 (3)
Yes, for some injections	22 (7)	27 (8)
Never	275 (88)	274 (85)
Data not available	0 (0)	10 (3)
Re‐used a needle or syringe after someone else^b^, *n* (%)		
No	29 (9)	38 (12)
Yes, always used disinfectant	251 (81)	241 (75)
Yes, sometimes or never used disinfectant	26 (8)	30 (9)
Data not available	5 (2)	12 (4)

^a^ Among participants who injected anytime in the current imprisonment.

^b^ Among participants who injected in the previous month in the current imprisonment.

### 3.2 HCV incidence

The median duration of follow‐up was 9 months (IQR: 5–18 months), with 669 participants (41%) having longer than 1 year of follow‐up. During 1818 person‐years of follow‐up, 111 incident HCV infections were detected, including 57 primary infections and 54 re‐infections. Five participants experienced two incident infection episodes during follow‐up. Among 57 participants with incident primary infection, 13 (23%) experienced spontaneous clearance, 30 (53%) progressed to chronic infection, and 14 (25%) did not have any follow‐up visit to assess the outcome. Among 54 re‐infections, 10 (19%) experienced spontaneous clearance, 28 (52%) progressed to chronic infection, and 16 (30%) were loss to follow‐up. The incidence rates of HCV infection, primary infection and re‐infection were 6.11/100 person‐years (95%CI: 5.07, 7.35), 4.60/100 person‐years (95%CI: 3.56, 5.96) and 9.34/100 person‐years (95%CI: 7.15, 12.19), respectively.

A total of 1150 participants stayed in prison for the entire follow‐up period, while 493 participants were released and re‐incarcerated during follow‐up. Among those staying in prison during the entire follow‐up period (991 person‐years follow‐up), the incidence rates of HCV infection, primary infection and re‐infection were 4.44/100 person‐years (95%CI: 3.31, 5.97), 2.57/100 person‐years (95%CI: 1.62, 4.09) and 8.92/100 person‐years (95%CI: 6.07, 13.10), respectively. Among those who were released and re‐incarcerated during the follow‐up (827 person‐years follow‐up), the incidence was higher. The incidence rates of HCV infection, primary infection and re‐infection in this group were 8.10/100 person‐years (95%CI: 6.37, 10.29), 7.21/100 person‐years (95%CI: 5.27, 9.87) and 9.76/100 person‐years (95%CI: 6.74, 14.14), respectively.

**FIGURE 2:**
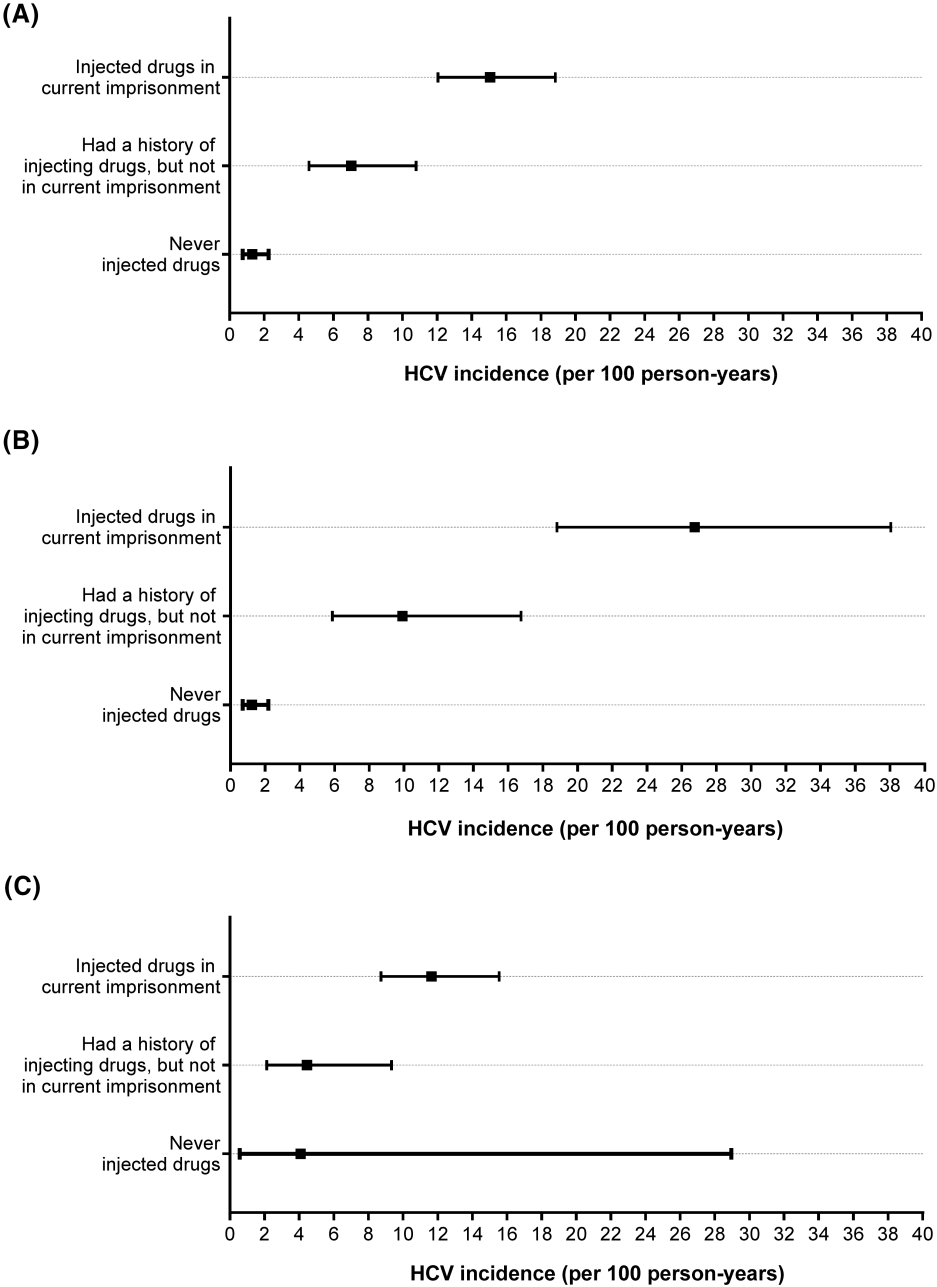
Incidence rate of HCV infection (A), primary infection (B), and re‐infection (C) among participants, by injecting drug use status at the beginning of the follow‐up.

**FIGURE 3:**
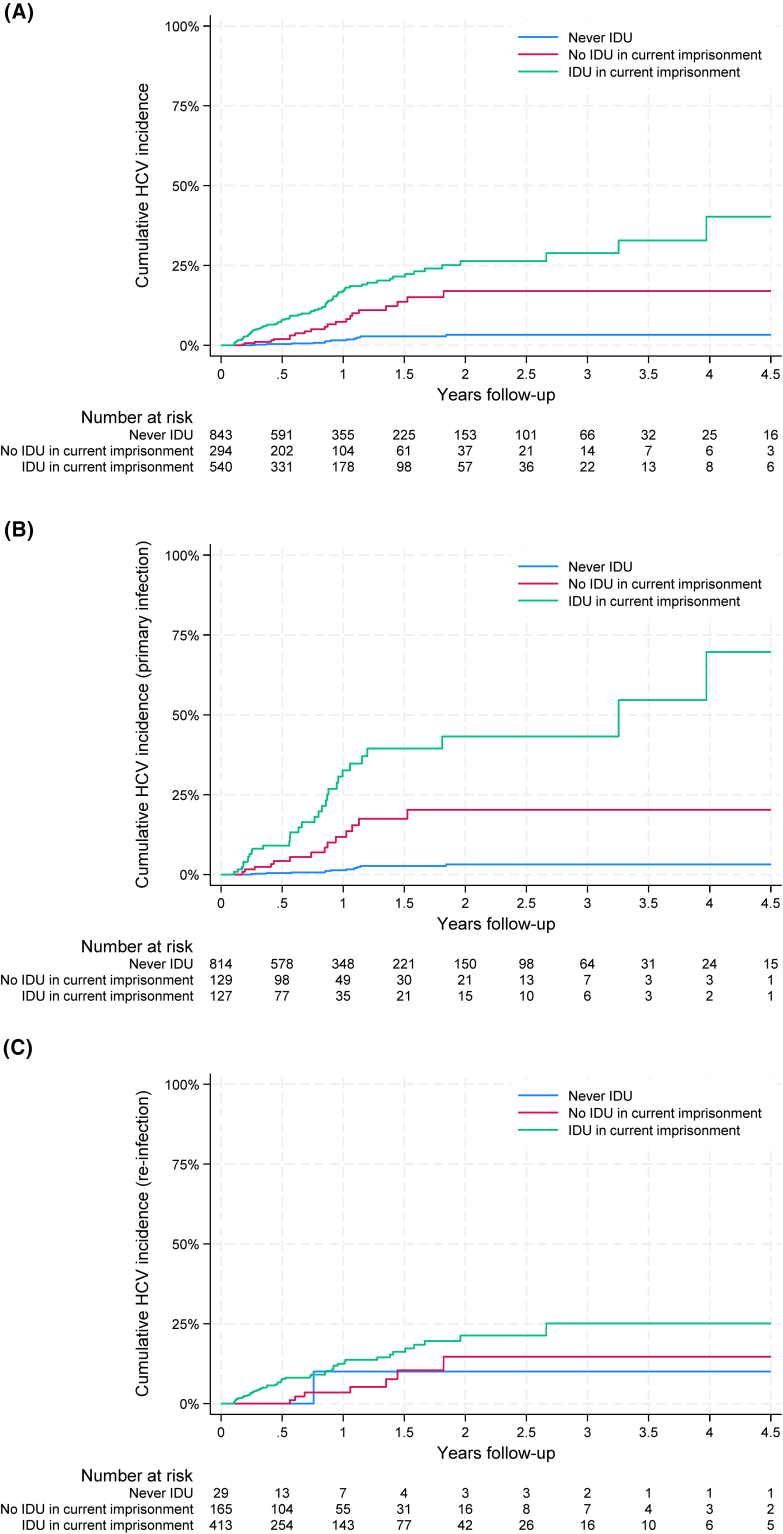
Cumulative incidence of HCV infection (A), primary infection (B) and re‐infection (C) among participants, by injecting drug use status at the beginning of the follow‐up. IDU: injecting drug us.

In an analysis stratified by participants' injecting drug use status at the beginning of the follow‐up, the incidence of HCV infection was highest among participants injecting drugs in the current imprisonment (15.07/100 person‐years, 95%CI: 12.05, 18.84), followed by those with a history of injecting drugs but not in the current imprisonment (7.03/100 person‐years, 95%CI: 4.59, 10.79). HCV incidence was low among participants who reported never injecting drugs (1.24/100 person‐years, 95%CI: 0.70, 2.17; Figure 2). The cumulative incidence was significantly different across the three groups (*p* < .001; Figure 3). Similarly, the highest incidence of primary HCV infection (26.75/100 person‐years, 95%CI: 18.81, 38.04) and HCV re‐infection (11.64/100 person‐years, 95%CI: 8.72, 15.54) was observed among participants injecting drugs in the current imprisonment (Figure 2).

### 3.3 Factors associated with HCV infection risk among all participants

Three adjusted Cox proportional hazards regression models were developed to address collinearity between gender and prison site and between injecting drug use and OAT (Table 2). In all three models, HCV risk was significantly lower in older participants, and higher among those who were released and re‐incarcerated during follow‐up. A decreased risk of HCV detection was also associated with a longer interval between HCV tests. In model 1, including injecting drug use status and prison site, HCV risk was significantly higher among participants reporting injecting drugs in the current imprisonment. Compared to those not injecting in the current imprisonment, the highest HCV risk was observed among those injecting in the previous month [adjusted hazard ration (HR): 8.01, 95%CI: 4.64, 13.82; Table 2]. In model 2, including injecting drug use and OAT status, people reporting injecting drugs in the current imprisonment who were not receiving OAT were at the highest risk of HCV compared with those who did not inject or receive OAT (adjusted HR: 10.55, 95%CI: 5.88, 18.92; Table 2). In model 3, including gender, no significant association was found between gender and HCV risk (Table 2).

**TABLE 2:** Cox Proportional Hazards models^a^ evaluating the factors associated with the risk of HCV infection among total analysis population.

	Unadjusted models		Adjusted model 1^b^	Adjusted model 2^c^	Adjusted model 3^d^
	Hazard ratio (95% CI)	*p* Value	Hazard ratio (95% CI)	Hazard ratio (95% CI)	Hazard ratio (95% CI)
Gender					
Male	1.00				1.00
Female	1.78 (1.12, 1.84)	.015			0.68 (0.38, 1.20)
Age at enrolment (per year increase)	0.92 (0.89, 0.94)	<.001	0.93 (0.91, 0.96)	0.93 (0.91, 0.96)	0.93 (0.90, 0.96)
Age at enrolment (year)					
25 years or younger	1.00				
25–35 years	0.66 (0.43, 1.01)	.053			
35–45 years	0.30 (0.17, 0.53)	<.001			
Older than 45 years	0.04 (0.01, 0.16)	<.001			
Aboriginal and or Torres Strait Islander					
No	1.00		1.00	1.00	1.00
Yes	2.05 (1.39, 3.01)	<.001	1.04 (0.68, 1.59)	1.09 (0.72, 1.67)	0.97 (0.63, 1.48)
Duration of stay in the current prison					
Up to 12 months	1.00		1.00	1.00	1.00
13–24 months	0.64 (0.37, 1.10)	.109	0.57 (0.32, 1.05)	0.66 (0.36, 1.21)	0.56 (0.31, 1.01)
25–36 months	0.53 (0.28, 1.02)	.056	0.73 (0.37, 1.47)	0.78 (0.39, 1.57)	0.61 (0.31, 1.21)
>36 months	0.34 (0.21, 0.55)	<.001	0.53 (0.30, 0.95)	0.60 (0.33, 1.07)	0.49 (0.28, 0.86)
Incarceration status during follow‐up					
Remained incarcerated	1.00		1.00	1.00	1.00
Released and re‐incarcerated	1.85 (1.25, 2.73)	.002	1.60 (1.03, 2.49)	1.56 (1.01, 2.43)	1.69 (1.09, 2.61)
Tattoo or piercing in the current imprisonment					
No	1.00		1.00	1.00	1.00
Yes	2.47 (1.55, 3.95)	<.001	1.30 (0.80, 2.13)	1.32 (0.81, 2.14)	1.25 (0.76, 2.04)
Injecting drug use status in the current imprisonment					
Not injected	1.00		1.00		1.00
Injected longer than 6 months ago	3.88 (1.75, 8.58)	.001	3.91 (1.68, 9.10)		3.93 (1.69, 9.11)
Injected in the previous 2–6 months	6.73 (3.04, 14.88)	<.001	6.33 (2.81, 14.22)		6.58 (2.93, 14.76)
Injected in the previous month	12.50 (7.47, 20.91)	<.001	8.01 (4.64, 13.82)		8.48 (4.91, 14.65)
Injecting drug use in the current imprisonment and OAT use					
Not injected; Not receiving OAT	1.00			1.00	
Not injected; Currently receiving OAT	3.02 (1.00, 9.12)	.049		3.59 (1.17, 11.02)	
Injected; Currently receiving OAT	2.48 (0.96, 6.40)	.060		2.35 (0.90, 6.17)	
Injected; Not receiving OAT	14.86 (8.52, 25.12)	<.001		10.55 (5.88, 18.92)	
HCV testing interval (per month increase)	0.69 (0.62, 0.76)	<.001	0.68 (0.62, 0.75)	0.67 (0.61, 0.74)	0.68 (0.62, 0.75)
Prison site at the last visit					
Lithgow	1.00		1.00	1.00	
Dillwynia	2.28 (1.28, 4.05)	.005	1.00 (0.50, 1.98)	0.99 (0.50, 1.97)	
Goulburn	2.02 (1.26, 3.24)	.004	1.98 (1.18, 3.31)	2.07 (1.22, 3.41)	
OMMPCC	0.59 (0.27, 1.27)	.177	0.96 (0.42, 2.17)	1.02 (0.45, 2.32)	

Abbreviation: OAT, Opioid agonist therapy.

^a^ All models were adjusted for HCV treatment scale‐up period. A total of 1731 person‐years of follow‐up with 98 incident events included in all adjusted models.

^b^ ‘Gender’ and ‘injecting drug use in the current imprisonment and OAT use’ were not included in the model due to collinearity with ‘Prison site at the last visit’ and ‘Injecting drug use status’, respectively.

^c^ ‘Injecting drug use status’ was replaced by ‘injecting drug use in the current imprisonment and OAT use’.

^d^ ‘Prison site at the last visit’ was replaced by ‘gender’.

The output of the models evaluating factors associated with the risk of HCV primary infection and re‐infection is included in Tables S1 and S2. Factors associated with primary HCV infection were almost similar with the overall models. Injecting drugs in the previous months, age and HCV testing interval were associated with HCV re‐infection.

### 3.4 Factors associated with HCV infection risk among participants injecting drugs in the past month

Participants who reported injecting drugs in the past month in prison had a total of 319 person‐years of follow‐up, with 59 incident HCV infection detected during follow‐up. A decreased HCV risk was associated with older age and longer HCV testing interval. HCV risk was also lower among people currently receiving OAT; however, the reduced risk was only statistically significant among those receiving high‐dosage OAT (Table 3). Compared with participants not sharing needle and syringe, an increased HCV risk was observed among those who shared needle and syringe without consistent use of disinfectant to clean equipment between injecting episodes (adjusted HR: 4.60, 95%CI: 1.35, 15.66; Table 3). The HCV risk was also higher among those who reported always using disinfectant to clean shared injecting equipment, but the difference was not statistically significant (Table 3).

**TABLE 3:** Cox Proportional Hazards models^a^ evaluating the factors associated with the risk of HCV infection among participants who injected drugs in the past month in prison.

	Unadjusted models		Adjusted model^b^
	Hazard Ratio (95% CI)	*p* Value	Hazard Ratio (95% CI)
Gender			
Male	1.00		
Female	1.24 (0.69, 2.23)	.464	
Age at enrolment (per year increase)	0.95 (0.91, 0.99)	.008	0.95 (0.91, 0.99)
Frequency of injecting			
Less frequently than once a week	1.00		1.00
1 to 6 days per week, not daily	1.61 (0.71, 3.64)	.256	1.42 (0.54, 3.70)
Once a day or more	1.71 (0.85, 3.46)	.134	1.31 (0.57, 2.99)
Substance injected (vs. not injecting that substance)			
Heroin, or other opiates	0.41 (0.15, 1.12)	.082	0.62 (0.20, 1.92)
Methadone, or buprenorphine	1.96 (0.84, 4.55)	.119	0.27 (0.06, 1.25)
Methamphetamine, or other stimulants	0.66 (0.39, 1.12)	.127	0.65 (0.32, 1.31)
Used a new sterile needle and syringe for injecting drugs			
Yes, for all injections	1.00		
Yes, for some injections	0.39 (0.06, 2.81)	.353	
Never	1.17 (0.28, 4.81)	.831	
Re‐used a needle or syringe after someone else had used it			
No re‐use of needle or syringe	1.00		1.00
Yes, but always used disinfectant	1.80 (0.71, 4.53)	.213	2.15 (0.81, 5.74)
Yes, sometimes or never used disinfectant	3.95 (1.28, 12.18)	.017	4.60 (1.35, 15.66)
Currently receiving opioid agonist therapy			
No	1.00		1.00
Yes, low dosage	0.43 (0.06, 3.13)	.407	0.33 (0.03, 3.85)
Yes, high dosage	0.23 (0.06, 0.96)	.044	0.11 (0.02, 0.80)
HCV testing interval (per month increase)	0.62 (0.54, 0.71)	<.001	0.63 (0.54, 0.73)
Prison site at the last visit			
Lithgow	1.00		
Dillwynia	1.35 (0.67, 2.72)	.395	
Goulburn	1.35 (0.73, 2.51)	.337	
OMMPCC	0.66 (0.24, 1.80)	.418	

^a^ All models were adjusted for HCV treatment scale‐up period.

^b^ 318 person‐years of follow‐up with 58 incident events included in the model; prison sites were considered as strata and stratified estimates were obtained.

## 4 DISCUSSION

This study demonstrated a high incidence of HCV infection in a large population of people in prison in Australia. In the total study population, recent injecting drug use in the prison (vs. no injecting) was associated with the greatest risk of HCV infection. Among people injecting in prison, those who used a shared needle or syringe and did not consistently use disinfectant for cleaning injecting equipment (vs. no sharing) were at higher risk while those who were receiving high‐dosage OAT (vs. no OAT) had a lower risk. These data reinforce the observation that people in prison are at a high HCV transmission risk, including in the DAA era. The findings of this study can inform design of targeted strategies for HCV prevention in the prison setting, including optimal provision of harm reduction (e.g. high‐coverage OAT, provision of needle and syringe programme) which have also additional health benefits for people who inject drugs in prison, such as HIV and overdose prevention.

In this study, the HCV incidence was 6.11/100 person‐years, with a higher rate (15.07/100 person‐years) among those who injected drugs during their current imprisonment. Another Australian study among incarcerated people with a history of injecting drug use reported an HCV incidence rate of 11/100 person‐years in the pre‐DAA era (2005–14).^10^ The lower HCV incidence in the SToP‐C study may relate to the broader study population (all people in prison regardless of drug use status) and the HCV treatment scale‐up implemented in the second phase of the study which did reduce incident infection. Previous analyses from this study demonstrated that HCV incidence rate in SToP‐C prisons was halved (from 8.31 to 4.35 per 100 person‐years) following HCV treatment scale‐up, indicating the important role of HCV treatment in controlling HCV transmission in the prisons.^12^

Prison‐based HCV treatment has made a major contribution to HCV treatment uptake in Australia, with 41% of national treatment initiations in 2021 provided through prison health services.^17^ However, high HCV incidence in the prisons may potentially undermine the success achieved by HCV treatment scale‐up. The HCV incidence among people with recent injecting in this study (15/100 person‐years) is higher than the incidence in people with recent injecting in the community (5/100 person‐years).^18^ The incidence of HCV re‐infection following DAA treatment in the SToP‐C study was 12.5/100 person‐years,^19^ which is also higher than the estimated incidence of 6/100 person‐years among people recently injecting drugs in the community.^20^ These data indicate a crucial need for enhanced HCV prevention strategies in Australian prisons. A modelling study projected that although the existing prison‐based interventions (e.g. OAT and HCV treatment) reduced HCV incidence, incarceration would contribute to 23% of new HCV infections in Australia by 2029.^21^ Internationally, there is also a major gap in recognition and investment on controlling the HCV epidemic in prisons. In a survey by the World Health Organisation in 2019 among 194 countries, 128 countries had a national viral hepatitis plan of which only 28 national plans referenced HCV testing, treatment or harm reduction for people in prison.^22^

People who inject drugs are over‐represented in prisons in most countries, including in Australia.^3^ In the SToP‐C study, 52% of participants had a history of injecting drug use, including 31% injecting drugs during the current imprisonment.^12^ Punitive drug policies are one of the major factors contributing to high incarceration rate of people who inject drugs. Among people who inject drugs, an estimated 58% globally and 53% in Australia had a history of incarceration.^4^ In 2021–22, drug‐related offences were the third most common principal offence in Australia, with 14% of offenders proceeded against by police having a drug‐related principal offence.^23^ In 2013, the Global Commission on Drug Policy released a report outlining the impact of strict law enforcements on HCV epidemics and advocated for non‐criminal response to drug use and drug possession for personal use.^24^

Our data identified a higher HCV incidence among participants who were released and re‐incarcerated compared to those continuously incarcerated during follow‐up. In people released and re‐incarcerated, we were not able to identify if the incident HCV was acquired in the community (after release) or in prison (before release or after re‐incarceration). Qualitative research has identified that re‐incarceration or transfer between prisons could disrupt access to injecting networks and ability to clean equipment which could increase HCV risk during the early period after re‐incarceration or transfer.^25^ On the contrary, a meta‐analysis of studies evaluating HCV incidence among people who inject drugs in community also identified an association between recent release from prison and increased risk of HCV infection.^26^ This finding is consistent with other evidence indicating a high likelihood of relapse to injecting drug use^27^, ^28^, ^29^ and increased injecting risk behaviour^29^, ^30^ following release, and suggests a consequent increased risk of HCV infection early in the period following release from prison. Interventions are therefore required for linkage to community‐based harm reduction services for people previously in prison, particularly soon after release.

Our findings demonstrated decreased HCV risk among participants receiving high‐, but not low‐dosage OAT. Previous data evaluating the effectiveness of prison‐based OAT in reducing HCV risk are varied.^10^, ^11^, ^31^, ^32^ One limitation in these studies is that they did not consider dosage of OAT in their analysis, a potentially important factor affecting injecting behaviours.^31^ Inadequate OAT dosage may not reduce craving for opioids and hence increase the likelihood that an individual will continue injecting while receiving OAT. Another study among people who inject drugs in the community also demonstrated that HCV risk was decreased only among people receiving high dosage that was perceived to be adequate.^33^

Access to OAT services in prison is a challenge internationally, with only 59 countries providing OAT in at least one prison in 2020.^34^ In several countries, including Australia, where prison‐based OAT is available, the coverage is suboptimal. Our findings identified that only 26% of participants who reported injecting drug use in the current imprisonment were receiving OAT, which is significantly lower than 40% recommended by the United Nations agencies.^35^ The limited capacity of OAT programmes in prison, and the logistics for maintaining continuity of OAT for those transferring between prisons, or released to the community are contributing factors to suboptimal OAT coverage and treatment interruptions.^36^ Long‐acting injectable depot‐buprenorphine with weekly or monthly dosing requirements is a safe and effective alternative option,^37^, ^38^ which can reduce the administrative and clinical burden and improve OAT coverage and continuity in prisons. However, it is still important for people who inject drugs to be able to choose their OAT medications. In a study in Australia among people who regularly used opioids, although most participants (68%) believed that long‐acting injectable depot‐buprenorphine was a good OAT option for them, a third of participants preferred other options.^39^

In Australian prisons, given that there is no access to needle and syringe programmes, the only other harm reduction option available for people who inject drugs is disinfectant for cleaning injecting equipment. Our findings showed that compared to people not sharing needles or syringes, the risk of HCV among those reporting sharing who always cleaned equipment with disinfectant between injecting episodes was not significantly different. The risk was only significantly higher among those who did not consistently use disinfectant to clean equipment. Although these findings may be taken to support the efficacy of using disinfectant in reducing HCV risk, it should be interpreted conservatively given the wide confidence interval around the hazard ratios. One challenge to the proper use of disinfectant is the complexity of the process, including three rinses with water followed by soaking in disinfectant for 5 min, and then three further rinses with water. In a survey in NSW prisons, only half (53%) of people who used disinfectant for cleaning needles and syringes followed the recommended process.^40^ In a qualitative study, people in prison reported restrictions on the time available to clean equipment so as not to come to the attention of the correctional staff, and as such were less likely to use disinfectant.^41^

In both analyses of factors associated with the risk of HCV primary infection and re‐infection, Aboriginal and or Torres Strait Islander people and those having tattoo or piercing in current imprisonment had significantly higher risk of HCV in unadjusted analyses, but not in adjusted analyses. It demonstrated that these variables were not independent risk factors for HCV transmission but were confounders in association of other risk factors (e.g. injecting drugs) and HCV.

This study had some limitations. Among participants enrolled, only 50% had a follow‐up assessment and were included in the analysis, reflective of high transition in prisoner population due to transfers between prisons or release to freedom. Most characteristics of participants with and without follow‐up were comparable, suggesting low chance of selection bias. However, a lower proportion of participants without follow‐up were receiving OAT. In the analysis population, only 41% had a follow‐up longer than 1 year. Our estimate of HCV re‐infection incidence could be an underestimation given that some re‐infection cases might have been spontaneously cleared before detection.^42^ In participants who were released and re‐incarcerated during follow‐up and had an incident HCV infection, we were not able to identify if the infection was acquired in the community or in prison.

In conclusion, this study demonstrated high HCV incidence in prison, particularly among those injecting drugs in prison. Among individuals with ongoing injecting drug use in prison, those receiving high‐dosage OAT were at decreased risk of HCV, while those using a shared needle or syringe without consistent disinfectant use to cleaning injecting equipment were at the higher risk of infection. High‐level HCV screening and treatment in prison should be complemented by enhanced HCV prevention strategies to control HCV transmission which would also have broader benefits on the health of people in prison who inject drugs.

## FUNDING INFORMATION

The Kirby Institute is funded by the Australian Government Department of Health and Ageing. The SToP‐C study is a partnership project involving the Kirby Institute, Justice Health and Forensic Mental Health Network, Corrective Services NSW, NSW Ministry of Health, NSW Users and AIDS Association, Hepatitis NSW and Gilead Sciences. GJD (2008276) and JG (1176131) are supported by NHMRC Investigator Grants. ARL is supported by an NHMRC Practitioner Fellowship. PV acknowledges support from the NIHR Health Protection Research Unit in Behavioural Science and Evaluation at the University of Bristol.

## CONFLICT OF INTEREST STATEMENT

GJD is a consultant or adviser for, and has received research grants from, AbbVie, Abbot Diagnostics, Gilead Sciences, Bristol Myers Squibb, Cepheid, GlaxoSmithKline, Merck, Janssen and Roche. ARL is a consultant/adviser and has received investigator‐initiated research grants from Gilead, AbbVie and Bristol‐Myers Squibb. JG is a consultant or adviser for, and has received research grants from AbbVie, bioLytical, Camurus, Cepheid, Gilead Sciences, Hologic and Indivor, and has received honoraria from AbbVie, Cepheid and Gilead Sciences. NM has received research grants from AbbVie and Gilead Sciences. PV has received research grants from Gilead Sciences. Other co‐authors had none to declare.

## Supporting information


Table S1

## Data Availability

The data that support the findings of this study are available on request from the corresponding author. The data are not publicly available due to privacy or ethical restrictions.
