# Supplementary material for: Incidence of hepatitis C virus infection in the prison setting: The SToP‐C study
Source: J Viral Hepat. 2023 Nov 7;31(1):21–34. doi: 10.1111/jvh.13895 (PMC10952254; doi:10.1111/jvh.13895)
Supplement: Supplementary file 1 — Table S1 [file JVH-31-21-s001.docx]

**Supplementary Table 1:** Cox Proportional Hazards models* evaluating the factors associated with the risk of HCV primary infection

|  | **Unadjusted models** | | **Adjusted model 1** | **Adjusted model 2†** |
| --- | --- | --- | --- | --- |
|  | **Hazard Ratio  (95% CI)** | **P  value** | **Hazard Ratio  (95% CI)** | **Hazard Ratio  (95% CI)** |
| **Gender** |  |  |  |  |
| Male | 1.00 |  |  |  |
| Female | 2.37 (1.23, 4.57) | 0.010 |  |  |
| **Age at enrolment (per year increase)** | 0.90 (0.87, 0.94) | <0.001 | 0.96 (0.93, 1.00) | 0.96 (0.92, 1.00) |
| **Aboriginal and or Torres Strait Islander** |  |  |  |  |
| No | 1.00 |  | 1.00 | 1.00 |
| Yes | 2.64 (1.52, 4.59) | 0.001 | 1.46 (0.77, 2.76) | 1.16 (0.61, 2.20) |
| **Duration of stay in the current prison** |  |  |  |  |
| Up to 12 months | 1.00 |  | 1.00 | 1.00 |
| 13-24 months | 0.42 (0.20, 0.92) | 0.027 | 0.93 (0.39, 2.21) | 0.96 (0.40, 2.29) |
| 25-36 months | 0.18 (0.06, 0.60) | 0.005 | 0.39 (0.11, 1.35) | 0.43 (0.12, 1.48) |
| >36 months | 0.14 (0.06, 0.29) | <0.001 | 0.27 (0.10, 0.71) | 0.35 (0.14, 0.90) |
| **Incarceration status during follow-up** |  |  |  |  |
| Remained incarcerated | 1.00 |  | 1.00 | 1.00 |
| Released and re-incarcerated | 2.74 (1.56, 4.83) | <0.001 | 2.26 (1.12, 4.55) | 2.12 (1.06, 4.28) |
| **Tattoo or piercing in current imprisonment** |  |  |  |  |
| No | 1.00 |  | 1.00 | 1.00 |
| Yes | 3.11 (1.61, 5.95) | 0.001 | 1.77 (0.87, 3.62) | 1.46 (0.75, 2.98) |
| **Injecting drug use status in the current imprisonment** |  |  |  |  |
| Not injected | 1.00 |  | 1.00 |  |
| Injected longer than 6 months ago | 19.16 (7.25, 50.66) | <0.001 | 14.45 (5.20, 40.17) |  |
| Injected in the previous 2-6 months | 16.94 (6.43, 44.65) | <0.001 | 20.77 (7.40, 58.33) |  |
| Injected in the previous month | 19.84 (10.09, 39.00) | <0.001 | 8.49 (4.04, 17.85) |  |
| **Injecting drug use in the current imprisonment and OAT use** |  |  |  |  |
| Not injected; Not receiving OAT | 1.00 |  |  | 1.00 |
| Not injected; Currently receiving OAT | 8.97 (1.98, 40.66) | 0.004 |  | 7.19 (1.52, 34.10) |
| Injected; Currently receiving OAT | 5.27 (0.68, 40.95) | 0.112 |  | 3.96 (0.46, 33.71) |
| Injected; Not receiving OAT | 24.48 (12.42, 48.26) | <0.001 |  | 13.59 (6.48, 28.49) |
| **HCV testing interval (per month increase)** | 0.81 (0.72, 0.91) | <0.001 | 0.76 (0.68, 0.85) | 0.76 (0.68, 0.85) |
| **Prison site at the last visit** |  |  |  |  |
| Lithgow | 1.00 |  | 1.00 | 1.00 |
| Dillwynia | 2.85 (1.27, 6.39) | 0.011 | 1.15 (0.45, 2.90) | 1.07 (0.41, 2.76) |
| Goulburn | 1.93 (1.00, 3.74) | 0.050 | 2.01 (0.96, 4.18) | 1.80 (0.85, 3.82) |
| OMMPCC | 0.48 (0.16, 1.49) | 0.207 | 0.73 (0.22, 2.38) | 0.73 (0.22, 2.42) |

OAT: Opioid agonist therapy

* all models were adjusted for HCV treatment scale-up period. A total of 1178 person-years of follow-up with 57 incident events included in the adjusted models

† "Injecting drug use status" was replaced by "injecting drug use in the current imprisonment and OAT use"

**Supplementary Table 2:** Cox Proportional Hazards models* evaluating the factors associated with the risk of HCV re-infection

|  | **Unadjusted models** | | **Adjusted model 1** | **Adjusted model 2†** |
| --- | --- | --- | --- | --- |
|  | **Hazard Ratio  (95% CI)** | **P  value** | **Hazard Ratio  (95% CI)** | **Hazard Ratio  (95% CI)** |
| **Gender** |  |  |  |  |
| Male | 1.00 |  |  |  |
| Female | 2.37 (1.23, 4.57) | 0.010 |  |  |
| **Age at enrolment (per year increase)** | 0.90 (0.87, 0.94) | <0.001 | 0.96 (0.93, 1.00) | 0.96 (0.92, 1.00) |
| **Aboriginal and or Torres Strait Islander** |  |  |  |  |
| No | 1.00 |  | 1.00 | 1.00 |
| Yes | 2.64 (1.52, 4.59) | 0.001 | 1.46 (0.77, 2.76) | 1.16 (0.61, 2.20) |
| **Duration of stay in the current prison** |  |  |  |  |
| Up to 12 months | 1.00 |  | 1.00 | 1.00 |
| 13-24 months | 0.42 (0.20, 0.92) | 0.027 | 0.93 (0.39, 2.21) | 0.96 (0.40, 2.29) |
| 25-36 months | 0.18 (0.06, 0.60) | 0.005 | 0.39 (0.11, 1.35) | 0.43 (0.12, 1.48) |
| >36 months | 0.14 (0.06, 0.29) | <0.001 | 0.27 (0.10, 0.71) | 0.35 (0.14, 0.90) |
| **Incarceration status during follow-up** |  |  |  |  |
| Remained incarcerated | 1.00 |  | 1.00 | 1.00 |
| Released and re-incarcerated | 2.74 (1.56, 4.83) | <0.001 | 2.26 (1.12, 4.55) | 2.12 (1.06, 4.28) |
| **Tattoo or piercing in current imprisonment** |  |  |  |  |
| No | 1.00 |  | 1.00 | 1.00 |
| Yes | 3.11 (1.61, 5.95) | 0.001 | 1.77 (0.87, 3.62) | 1.46 (0.75, 2.98) |
| **Injecting drug use status in the current imprisonment** |  |  |  |  |
| Not injected | 1.00 |  | 1.00 |  |
| Injected longer than 6 months ago | 19.16 (7.25, 50.66) | <0.001 | 14.45 (5.20, 40.17) |  |
| Injected in the previous 2-6 months | 16.94 (6.43, 44.65) | <0.001 | 20.77 (7.40, 58.33) |  |
| Injected in the previous month | 19.84 (10.09, 39.00) | <0.001 | 8.49 (4.04, 17.85) |  |
| **Injecting drug use in the current imprisonment and OAT use** |  |  |  |  |
| Not injected; Not receiving OAT | 1.00 |  |  | 1.00 |
| Not injected; Currently receiving OAT | 8.97 (1.98, 40.66) | 0.004 |  | 7.19 (1.52, 34.10) |
| Injected; Currently receiving OAT | 5.27 (0.68, 40.95) | 0.112 |  | 3.96 (0.46, 33.71) |
| Injected; Not receiving OAT | 24.48 (12.42, 48.26) | <0.001 |  | 13.59 (6.48, 28.49) |
| **HCV testing interval (per month increase)** | 0.81 (0.72, 0.91) | <0.001 | 0.76 (0.68, 0.85) | 0.76 (0.68, 0.85) |
| **Prison site at the last visit** |  |  |  |  |
| Lithgow | 1.00 |  | 1.00 | 1.00 |
| Dillwynia | 2.85 (1.27, 6.39) | 0.011 | 1.15 (0.45, 2.90) | 1.07 (0.41, 2.76) |
| Goulburn | 1.93 (1.00, 3.74) | 0.050 | 2.01 (0.96, 4.18) | 1.80 (0.85, 3.82) |
| OMMPCC | 0.48 (0.16, 1.49) | 0.207 | 0.73 (0.22, 2.38) | 0.73 (0.22, 2.42) |

OAT: Opioid agonist therapy

* all models were adjusted for HCV treatment scale-up period. A total of 564 person-years of follow-up with 50 incident events included in the adjusted models

† "Injecting drug use status" was replaced by "injecting drug use in the current imprisonment and OAT use"
